# Tissue-specific role of RHBDF2 in cutaneous wound healing and hyperproliferative skin disease

**DOI:** 10.1186/s13104-017-2899-8

**Published:** 2017-11-07

**Authors:** Vishnu Hosur, Bonnie L. Lyons, Lisa M. Burzenski, Leonard D. Shultz

**Affiliations:** The Jackson Laboratory, 600 Main Street, Bar Harbor, ME 04609 USA

**Keywords:** Tylosis, RHBDF2, Amphiregulin, MRL/MpJ, Wound healing

## Abstract

**Objective:**

Gain-of-function (GOF) mutations in *RHBDF2* cause tylosis. Patients present with hyperproliferative skin, and keratinocytes from tylosis patients’ skin show an enhanced wound-healing phenotype. The curly bare mouse model of tylosis, carrying a GOF mutation in the *Rhbdf2* gene (*Rhbdf2* ^*cub*^), presents with epidermal hyperplasia and shows accelerated cutaneous wound-healing phenotype through enhanced secretion of the epidermal growth factor receptor family ligand amphiregulin. Despite these advances in our understanding of tylosis, key questions remain. For instance, it is not known whether the disease is skin-specific, whether the immune system or the surrounding microenvironment plays a role, and whether mouse genetic background influences the hyperproliferative-skin and wound-healing phenotypes observed in *Rhbdf2* ^*cub*^ mice.

**Results:**

We performed bone marrow transfers and reciprocal skin transplants and found that bone marrow transfer from C57BL/6 (B6)-*Rhbdf2* ^*cub/cub*^ donor mice to B6 wildtype recipient mice failed to transfer the hyperproliferative-skin and wound-healing phenotypes in B6 mice. Furthermore, skin grafts from B6 mice to the dorsal skin of B6-*Rhbdf2* ^*cub/cub*^ mice maintained the phenotype of the donor mice. To test the influence of mouse genetic background, we backcrossed *Rhbdf2* ^*cub*^ onto the MRL/MpJ strain and found that the hyperproliferative-skin and wound-healing phenotypes caused by the *Rhbdf2* ^*cub*^ mutation persisted on the MRL/MpJ strain.

## Introduction

Tylosis, a genetic disease characterized by hyperproliferation of skin in the palms and soles, loss of hair, and oral leukoplakia [1], is caused by gain-of-function (GOF) mutations (p.I186T, p.P189L, and p.D188N) in the human rhomboid family protein RHBDF2 [1, 2]. We recently showed that a spontaneous deletion of exons 2 through 6 in the *Rhbdf2* gene in C57BL/6J mice that underlies the curly bare mutation (*Rhbdf2* ^*cub*^) yields a mutant protein lacking the cytosolic N-terminal domain (ΔN-RHBDF2) [3, 4]. We also showed that this mutant protein specifically enhances secretion of the epidermal growth factor receptor (EGFR) family ligand amphiregulin (AREG) in various tissues, including skin and intestine [3]. Notably, *Rhbdf2* ^*cub*^ mice exhibit complete hair loss and rapid ear-wound healing (assessed via ear-punch hole closure).

Additionally, we developed a mouse model of human tylosis by using CRISPR/Cas9-mediated gene editing to generate mice carrying the human tylosis disease mutation p.P189L (p.P159L in mice). Consistent with the *Rhbdf2* ^*cub/cub*^ phenotype, *Rhbdf2* ^*P159L/P159L*^ mice exhibited severe epidermal hyperplasia and hyperkeratosis, and showed accelerated wound healing [5]. To test whether high AREG levels mediate the hyperproliferative-skin and wound-healing phenotypes, we crossed *Rhbdf2* ^*P159L/P159L*^ mice with *Areg*-null mice (B6.Cg-*Areg* ^*Mcub*^ *Rhbdf2* ^*cub*^/J, hereafter referred to as *Areg* ^−*/*−^ mice), and found that *Rhbdf2* ^*P159L/P159L*^ *Areg* ^−*/*−^ mice exhibited neither the hyperactive EGFR nor the hair-loss phenotype, indicating that increased AREG levels alone mediate the hyperproliferative-skin and wound-healing phenotypes [5]. Collectively, these studies suggest that AREG is a functional driver of tylosis; however, the role of the immune system and the effect of the genetic background on the tylosis phenotype remain unknown. Here, we tested the hypothesis that the hyperproliferative-skin and rapid wound-healing phenotypes observed in tylosis is tissue-specific and persists independently of the immune system. Using genetic approaches, bone marrow transplants, and reciprocal skin grafts, we show that a tissue-specific function of RHBDF2 rather than the surrounding microenvironment or the immune system underlies this skin disease.

## Main text

### Methods

#### Animals

All animal procedures were performed in accordance with the guidelines of the Animal Care and Use Committee of The Jackson Laboratory, and conformed to regulations in the Guide for the Care and Use of Laboratory Animals (Institute of Laboratory Animal Resources, National Research Council, National Academy of Sciences, 8th edition, 2011). Euthanasia was performed in a way consistent with the 2013 recommendations of the American Veterinary Medical Association (AVMA) *Guidelines on Euthanasia*. Mice were bred and maintained under modified barrier conditions at The Jackson Laboratory. To generate MRL/MpJ-*Rhbdf2* ^*cub/cub*^ congenic mice, C57BL/6J-*Rhbdf2* ^*cub/cub*^ mice were backcrossed onto the MRL/MpJ strain background for more than 20 generations. The following primer pairs were used to genotype *Rhbdf2* ^*cub/cub*^ mice: *Rhbdf2* ^*cub*^ forward: TGT GGA ATA CCC CCA AAG AAG C; *Rhbdf2* ^*cub*^ reverse: ATA ACC CAT AGC AGA GGA GGC G; *Rhbdf2* wildtype forward: TGC CCA CAC CGT ATC TGT TCT G; *Rhbdf2* wildtype reverse: GTT TTG GAG ACT CAGTGC CCT G. B6.Cg-*Areg* ^*Mcub*^ *Rhbdf2* ^*cub*^/J mice are referred to as *Rhbdf2* ^*cub/cub*^ *Areg* ^−*/*−^ mice.

#### Histology

Performed as described previously [3].

#### Bone-marrow chimeras and ADVIA cell counts

To generate bone-marrow chimeras, two groups of recipient male B6 mice, 15 mice/group, were first irradiated with a single lethal dose of 1000 cGy delivered by a Shepard Mark I irradiator containing^137^Cs (J. L. Shepard and Assoc., San Fernando, CA). Then the first group received bone marrow collected from femurs of male B6 mice, and the second group received bone marrow from the femurs of male B6-*Rhbdf2* ^*cub/cub*^ mice. For all mice, engraftment was via intravenously injection of 3 × 10^6^ cells in 200 μLs of sterile RPMI 1640 medium. Post-engraftment a complete blood count (Siemens ADVIA 120 Hematology System) was run on recipient mice to test for any differences in the rates of bone marrow engraftment. Twelve weeks post-engraftment, wound-healing assays were performed by punching 2-mm through-and-through holes in both the right and left ears of recipient mice using a surgical ear punch device (Napox KN-292B; Natsume Seisakusho Co.) [3]. Wound closure was assessed by measuring the percentage of ear-hole closure after 4 weeks of wounding in recipient mice.

#### Reciprocal skin grafting

Mice were anesthetized with tribromoethanol (400 mg/kg IP) and an analgesic (buprenorphine 0.05 mg/kg SC) was administered. The fur was removed from the dorsal-lateral thorax with clippers and the surgical site was disinfected with 10% povidone-iodine alternating with 70% ethanol. An oblong 8 × 10 mm full thickness section of skin was excised bilaterally with curved scissors. The skin sections were placed in a petri dish containing cold sterile saline. Once grafts were excised from paired mice for reciprocal transplantation the skin grafts were fitted into the recipients sites. Scissors were used to trim the graft as necessary and the graft was rotated such that fur growth on the graft would be in the direction opposite to the recipient’s fur. A small amount of tissue adhesive was used to secure the grafts in position. A section of sterile non-adhesive gauze pad was placed over the grafts and a self-adhesive bandage (VetRap) was placed around the thorax. Mice were examined daily and the bandage removed at 5 days after surgery.

#### Isolation of primary fibroblasts and keratinocytes

Performed as described previously [5].

### Results

#### Bone marrow transplants fail to confer the wound-healing phenotype to slow-healer B6 mice

To test whether the regenerative phenotype could be transferred from B6.*Rhbdf2* ^*cub/cub*^ to B6 slow-healer mice, we performed bone-marrow transfer experiments. Two-mm through-and-through holes were punched into the ears of recipient mice and analyzed. B6.*Rhbdf2* ^*cub/cub*^ mice showed rapid wound healing, with up to 95% ear-hole closure 4 weeks post-wounding. Both B6 bone marrow recipient groups—mice receiving bone marrow from femurs of B6 mice and mice receiving bone marrow from femurs of B6.*Rhbdf2* ^*cub/cub*^ mice—showed no difference in ear punch hole diameter (data not shown). To test whether the recipient mice showed any differences in the rates of recovery from lethal irradiation, we performed a complete blood count analysis and observed similar rates of recovery in the bone marrow from lethal irradiation (data not shown). Thus, bone-marrow engraftment from B6.*Rhbdf2* ^*cub/cub*^ donor mice into slow-healer B6 recipient mice failed to transfer the cutaneous rapid wound-healing phenotype, indicating that the immune system does not regulate the wound-healing phenotype observed in *Rhbdf2* ^*cub/cub*^ mice.

#### Reciprocal skin grafts indicate tissue-specific function of *Rhbdf2*^*cub*^

**Fig. 1:**
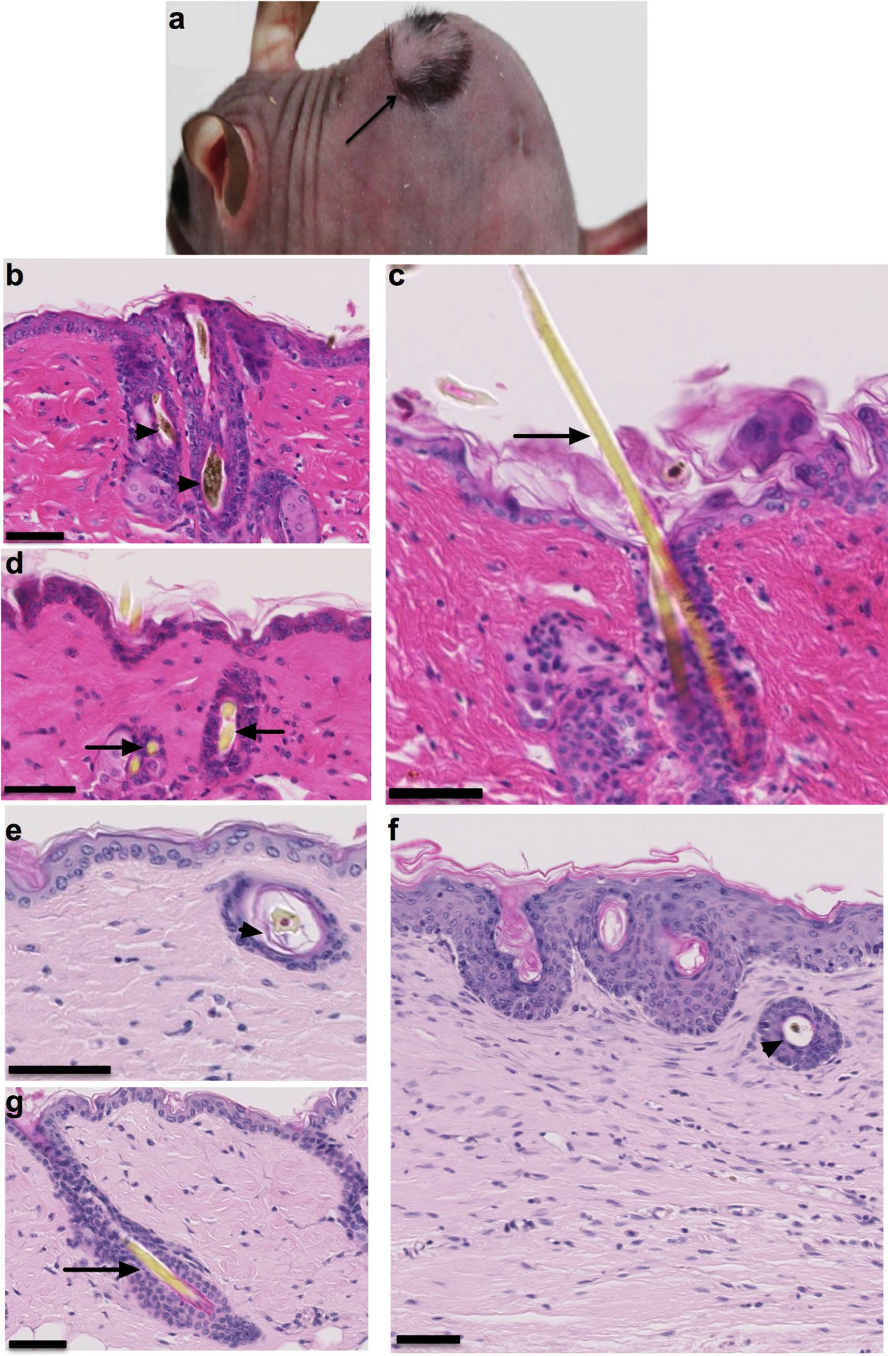
Reciprocal skin grafts. **a** Representative image of a recipient mouse with a skin graft, showing a recipient B6-*Rhbdf2* ^*cub/cub*^ mouse with a skin graft from B6-*Rhbdf2* ^+*/*+^ mouse showing retention of hair growth at 12 weeks post-skin graft. **b** H&E-stained skin section of a female B6-*Rhbdf2* ^*cub/cub*^ mouse, showing follicular dystrophy (indicated by arrowhead). Scale bar: 50 μm. **c** H&E-stained skin section of a female B6-*Rhbdf2* ^*cub/cub*^ mouse with a B6-*Rhbdf2* ^+*/*+^ donor skin graft displaying normal hair growth with no evidence of follicular dystrophy (arrows). Scale bar: 50 μm. **d** H&E-stained skin section of a female B6-*Rhbdf2* ^*cub/cub*^ mouse with a B6-*Rhbdf2* ^*cub/cub*^ *Areg* ^−*/*−^ donor skin graft displaying normal hair growth with no evidence of follicular dystrophy (arrows). Scale bar: 50 μm. **e** H&E-stained skin section of a female B6-*Rhbdf2* ^+*/*+^ mouse with a B6-*Rhbdf2* ^*cub/cub*^ donor skin graft displaying follicular dystrophy (arrowhead); Scale bar: 50 μm. **f** H&E-stained skin section of a female B6-*Rhbdf2* ^*cub/cub*^ *Areg* ^−*/*−^ mouse with no skin graft, displaying normal hair growth (arrow). Scale bar: 50 μm. **g** H&E-stained skin section of a female B6-*Rhbdf2* ^*cub/cub*^ *Areg* ^−*/*−^ mouse with a B6-*Rhbdf2* ^*cub/cub*^ donor skin graft, displaying follicular dystrophy (arrowhead). Scale bar: 50 μm

To test whether tissue-specific or non-tissue-specific effects of *Rhbdf2* ^*cub*^ cause the hyperproliferative skin phenotype, reciprocal skin grafts were performed by placing full-thickness skin grafts from littermate control (B6 wildtype) mice onto the dorsal skin of B6-*Rhbdf2* ^*cub/cub*^ mice and skin grafts from B6-*Rhbdf2* ^*cub/cub*^ mice onto B6-wildtype mice. In addition, skin grafts from B6-*Rhbdf2* ^*cub/cub*^ *Areg* ^−*/*−^ mice, which present a full but wavy hair coat [3], were transplanted onto B6-*Rhbdf2* ^*cub/cub*^ mice. Because all mice were congenic on the C57BL/6 J background, all were histocompatible. After 12 weeks, the skin grafts maintained the phenotype of the donor animal (Fig. 1a), suggesting that the phenotype was tissue-specific and persisted independently of the surrounding microenvironment. Histological examination of hematoxylin and eosin (H&E)-stained slides revealed follicular dystrophy in *Rhbdf2* ^*cub/cub*^ mice (Fig. 1b); however, *Rhbdf2* ^*cub/cub*^ mice receiving skin grafts from either *Rhbdf2* ^+*/*+^ (Fig. 1c), or *Rhbdf2* ^*cub/cub*^ *Areg* ^−*/*−^ mice (Fig. 1d), retained the skin phenotype of the donor animal—no evidence of follicular dystrophy and normal hair growth. Additionally, skin grafts from *Rhbdf2* ^*cub/cub*^ mice exhibited follicular dystrophy following transplantation onto *Rhbdf2* ^+*/*+^ mice (Fig. 1e). Lastly, skin grafts from *Rhbdf2* ^*cub/cub*^ mice engrafted onto *Rhbdf2* ^*cub/cub*^ *Areg* ^−*/*−^ mice (Fig. 1f) resulted in maintenance of the donor skin hairloss phenotype (Fig. 1g). Together, these results indicate that a tissue-specific effect of *Rhbdf2* ^*cub*^ underlies the tylosis phenotype.

#### *Rhbdf2* gain-of-function accelerates cutaneous wound healing in MRL/MpJ ‘healer’ mice

**Fig. 2:**
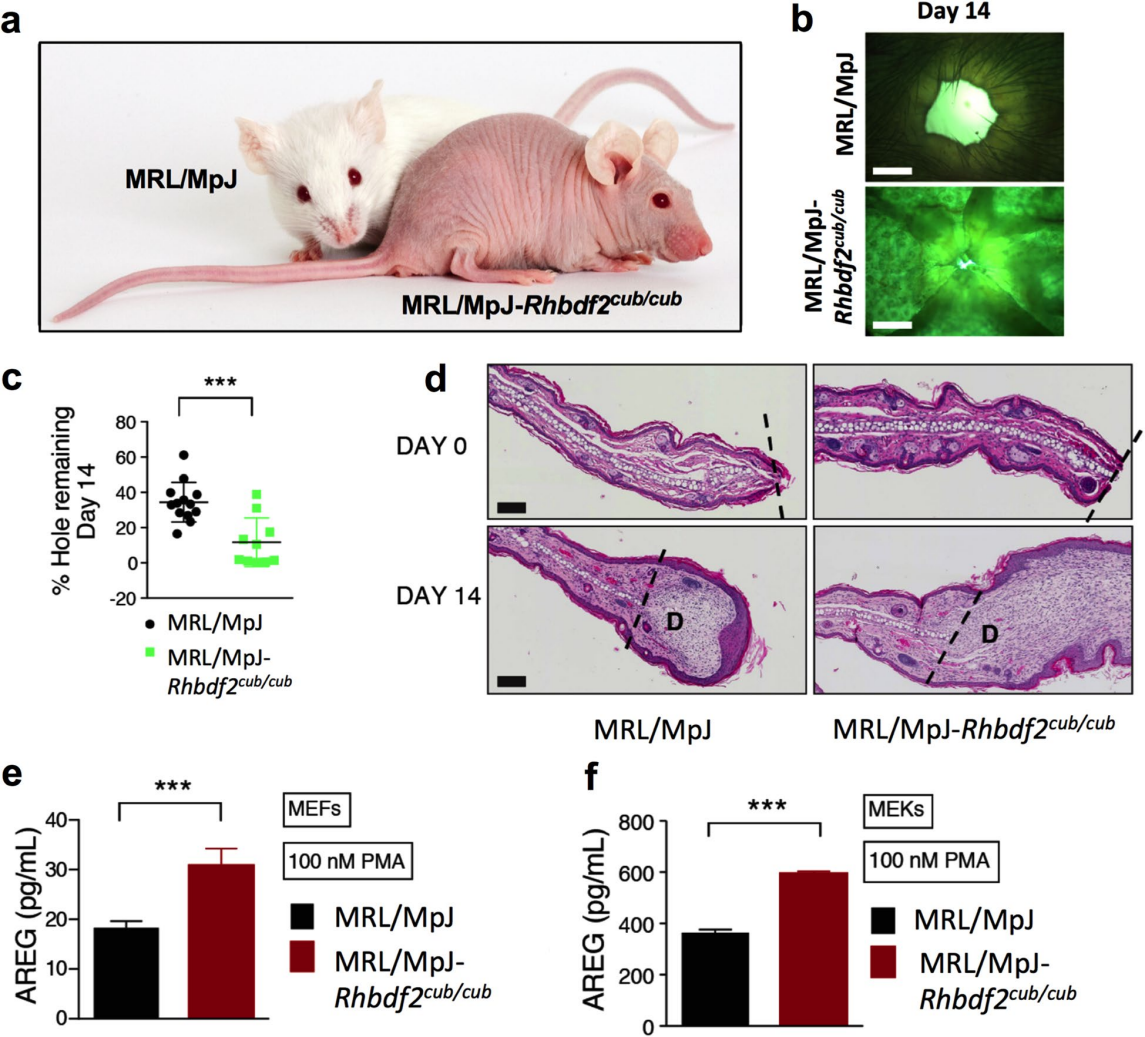
The *Rhbdf2* ^*cub/cub*^ mutation accelerates wound healing in MRL/MpJ “healer mice”. **a** The *Rhbdf2* ^*cub/cub*^ mutation leads to complete hair loss in the MRL/MpJ strain. **b** Representative images of regenerating ear tissue in 8-week-old female MRL/MpJ and MRL/MpJ-*Rhbdf2* ^*cub/cub*^ mice at 14 days post-wounding; (magnification = 4 ×; scale bars = 1 mm). **c** Quantification determined by measuring the area of the ear-holes shown in **b**; MRL/MpJ (n = 7), MRL/MpJ-*Rhbdf2* ^*cub/cub*^ mice (n = 5). **d** H&E-stained cross-sections of ears from MRL/MpJ and MRL/MpJ-*Rhbdf2* ^*cub/cub*^ mice at 0 and 14 days post-wounding. The dashed line indicates excision site; magnification = ×10, scale bars = 100 μm; (D) degree of proliferation. **e**, **f** ELISA quantitation of AREG levels in the supernatants of cultured MEFs and MEKs isolated from MRL/MpJ and MRL/MpJ-*Rhbdf2* ^*cub/cub*^ mice, in response to stimulation with 100 nM PMA

To test the influence of genetic background on the *Rhbdf2* ^*cub*^ wound-healing phenotype, we examined whether the *Rhbdf2* ^*cub*^ mutation can accelerate wound healing in MRL/MpJ ‘healer’ mice, which have the capacity to regenerate ear hole-punch wounds without scarring [6]. To create a true congenic strain by moving the *Rhbdf2* ^*cub/cub*^ mutation onto the MRL/MpJ background, B6-*Rhbdf2* ^*cub*^ mice were backcrossed onto the MRL/MpJ background for more than 20 generations (Fig. 2a). We punched 2-mm through-and-through holes in the ears of MRL/MpJ and MRL-*Rhbdf2* ^*cub/cub*^ mice, and observed that the *Rhbdf2* ^*cub*^ mutation significantly accelerated wound healing in MRL/MpJ mice (Fig. 2b, c). Cross-sections of ears from MRL/MpJ and MRL/MpJ-*Rhbdf2* ^*cub/cub*^ mice taken at 14 day post-wounding revealed an extensive degree of proliferation in the ears of MRL-*Rhbdf2* ^*cub/cub*^ mice (Fig. 2d). Additionally, MRL/MpJ-*Rhbdf2* ^*cub/cub*^ mouse embryonic fibroblasts (MEFs) (Fig. 2e) and mouse embryonic keratinocytes (MEKs) (Fig. 2f) produced significantly higher levels of AREG compared with MRL/MpJ wildtype MEFs and MEKs after stimulation with phorbol-12-myristate-13-acetate (PMA).

**Fig. 3:**
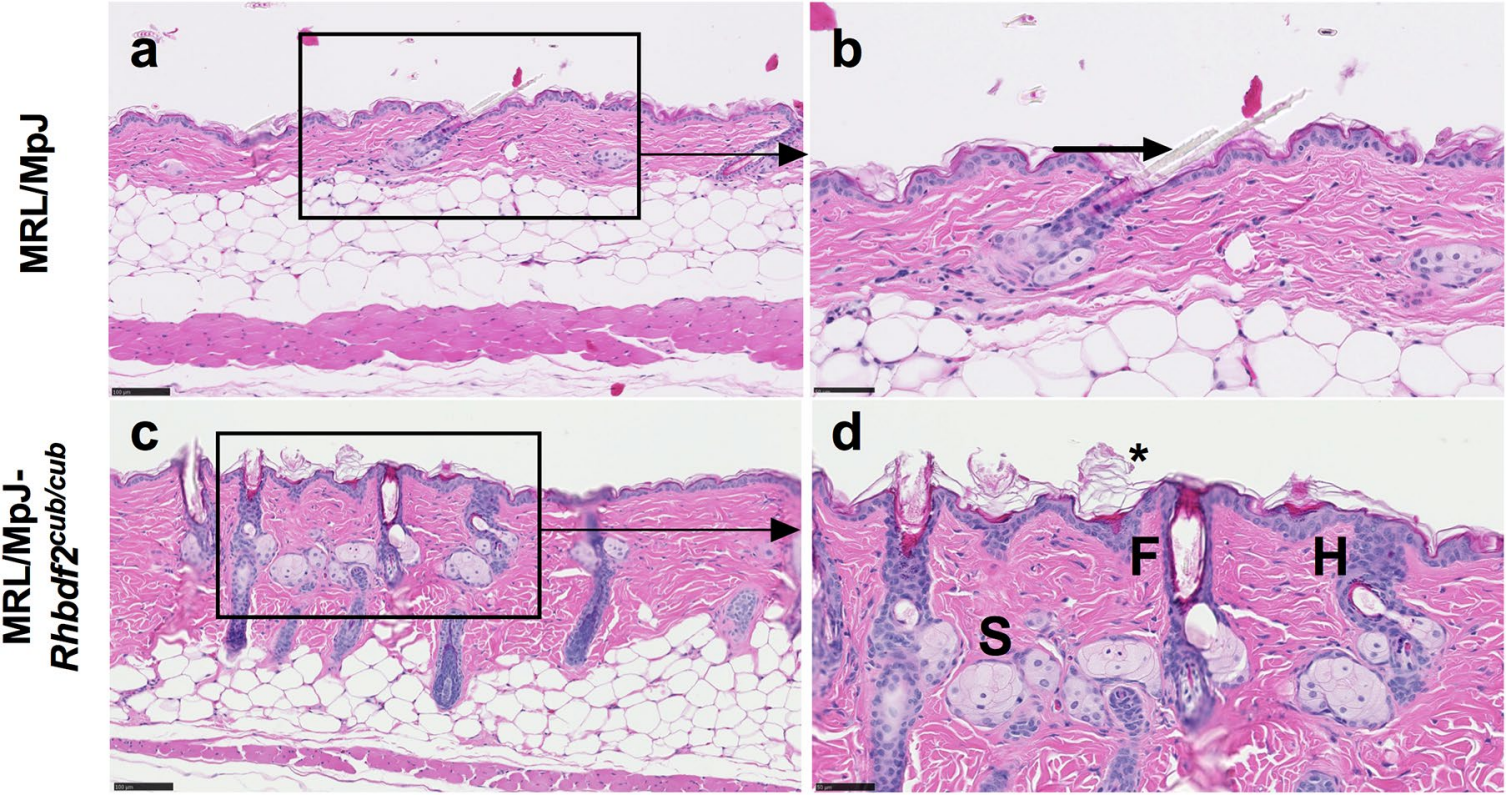
MRL/MpJ-*Rhbdf2* ^*cub/cub*^ mice exhibit the tylosis skin phenotype. **a**, **b** H&E-stained skin section of a female MRL/MpJ mouse showing normal hair growth (arrow); scale bar = 100 μm (**a**); scale bar = 50 μm (**b**). **c**, **d** H&E-stained skin section of a female MRL/MpJ-*Rhbdf2* ^*cub/cub*^ mouse showing follicular dystrophy (F), enlarged sebaceous glands (S), hyperplasia (H), and hyperkeratosis (asterisk); scale bar = 100 μm (**c**); scale bar = 50 μm (**d**)

Additionally, we examined the skin phenotype of MRL/MpJ-*Rhbdf2* ^*cub/cub*^ mice, and observed that MRL/MpJ mice homozygous for the *Rhbdf2* ^*cub*^ allele, but not those heterozygous for the *Rhbdf2* ^*cub*^ allele, presented with complete hair loss. Moreover, histological analysis of H&E-stained skin sections of female MRL/MpJ-*Rhbdf2* ^+*/*+^ (Fig. 3a, b) and MRL/MpJ-*Rhbdf2* ^*cub/cub*^ mice (Fig. 3c, d) mice revealed follicular dystrophy (F), enlarged sebaceous glands (S), hyperplasia (H), and hyperkeratosis (asterisk) in MRL/MpJ-*Rhbdf2* ^*cub/cub*^ mice (Fig. 3c, d), but not in MRL/MpJ-*Rhbdf2* ^+*/*+^; this phenotype in MRL/MpJ-*Rhbdf2* ^*cub/cub*^ mice is similar to the *Rhbdf2* ^*cub/cub*^ phenotype observed on the B6 strain background [3]. Collectively, these data suggest that the *Rhbdf2* ^*cub*^ mutation accelerates cutaneous wound healing in MRL/MpJ ‘healer’ mice and that the hyperproliferative-skin and wound-healing phenotypes persist independently of the mouse inbred strain background.

### Discussion

Tylosis, a form of palmoplantar keratoderma, is a hyperproliferative skin disease associated with increased risk of developing esophageal cancer [7, 8]. Currently there is no cure for tylosis or tylosis-associated carcinomas. Despite recent advances in the understanding of the genetic and biological factors underlying tylosis [9], including discoveries made by our group using the *Rhbdf2* ^*cub*^ strain, a mouse model of tylosis that shows an accelerated wound-healing phenotype, key questions remain. In this study we investigated whether the role of the *Rhbdf2* ^*cub*^ mutation in tylosis is tissue-specific or non-tissue-specific; and whether the immune system or the surrounding microenvironment plays a role in tylosis. To determine whether the immune system or the surrounding microenvironment plays a role in tylosis and whether the role of the *Rhbdf2* ^*cub*^ mutation in tylosis is tissue-specific or non-tissue-specific, we performed bone-marrow transfer and reciprocal skin graft experiments. Bone marrow data indicate that the immune system does not regulate the regenerative phenotype observed in *Rhbdf2* ^*cub/cub*^ mice, and reciprocal skin grafts data suggest that the tylosis phenotype is tissue-specific and persists independently of the surrounding microenvironment.

In addition to yielding the new information on tylosis summarized above, this study provides data on the effect of genetic background on the tylosis phenotype. We backcrossed the *Rhbdf2* ^*cub*^ mutation onto the MRL/MpJ ‘healer’ strain background and examined the wound-healing phenotype in congenic MRL/MpJ-*Rhbdf2* ^*cub/cub*^ mice. The accelerated wound-healing phenotype, epidermal hyperplasia, and the loss of hair phenotypes were retained in this congenic strain, suggesting that the tylosis phenotype persists independently of mouse strain background.

Our study also sheds light on the cell types responsible for tylosis; currently, the primary cell types responsible for tylosis are not known. Results of the current study, together with previous findings in our laboratory, will be helpful in laying the foundation for our future studies. Our previous studies using *Rhbdf2* ^*cub*^ mice provided information on the possible role of pro-inflammatory cytokine tumor necrosis factor alpha (TNFA) [3]. RHBDF2 was shown by our group and others to be essential for stimulated secretion of TNFA; *Rhbdf2* knockout mice fail to secrete TNFA in response to bacterial endotoxin lipopolysaccharide (LPS) [3, 10–12]. Thus, it is possible that GOF mutations in *RHBDF2,* such as *Rhbdf2* ^*cub/cub*^ and *Rhbdf2* ^*P159L/P159L*^, influence TNFA secretion, contributing to tylosis. However, our recent gene-deletion studies, in which we deleted AREG and observed restoration of the normal skin phenotype in both *Rhbdf2* ^*cub/cub*^ [3] and *Rhbdf2* ^*P159L/P159L*^ mice [5], strongly argue against a role for TNFA in tylosis.

Several lines of evidence point to keratinocytes as the primary cell type responsible for tylosis disease. First, keratinocytes are the major cell type producing AREG in skin [13, 14], and RHBDF2 is predominantly expressed in the skin [3]. Second, keratinocytes from tylosis patients show a wound-healing phenotype—accelerated proliferation and migration through constitutive activation of EGFR signaling [1]. Together, these previous observations and results from our present study provide valuable background information for future studies aimed at testing keratinocyte-specific effects of *RHBDF2* in tylosis in skin tissue. In addition, based on our previous findings showing that increased AREG levels mediate the *Rhbdf2* ^*cub*^ phenotype [3], and the findings of the present study, we propose that tylosis therapies should be targeted toward inhibition of AREG specifically in the skin.

## Limitations

The primary cell types responsible for tylosis are not known. We plan to carry out future studies to identify the responsible cell types, and as a key component of this we are currently developing conditional *Rhbdf2* ^*cub*^ mice.

## Abbreviations

AREG: amphiregulin
B6: C57BL/6
EGFR: epidermal growth factor receptor
GOF: gain-of-function
LPS: lipopolysaccharide
MEFs: mouse embryonic fibroblasts
MEKs: mouse embryonic keratinocytes
ΔN-RHBDF2: RHBDF2 lacking the cytosolic N-terminal domain
TNFA: tumor necrosis factor alpha
